# Learning processes in hierarchical pairs regulate entire gene expression in cells

**DOI:** 10.1038/s41598-022-10998-z

**Published:** 2022-05-09

**Authors:** Tomoyuki Yamaguchi

**Affiliations:** Research Institute, Nozaki Tokushukai Hospital, Daito City, Osaka 574-0074 Japan

**Keywords:** Stochastic networks, Gene expression, Complexity

## Abstract

Expression of numerous genes is precisely controlled in a cell in various contexts. While genetic and epigenetic mechanisms contribute to this regulation, how each mechanism cooperates to ensure the proper expression patterns of the whole gene remains unclear. Here, I theoretically show that the repetition of simple biological processes makes cells functional with the appropriate expression patterns of all genes if the inappropriateness of current expression ratios is roughly fed back to the epigenetic states. A learning pair model is developed, in which two factors autonomously approach the target ratio by repeating two stochastic processes; competitive amplification with a small addition term and decay depending on the difference between the current and target ratios. Furthermore, thousands of factors are self-regulated in a hierarchical-pair architecture, in which the activation degrees competitively amplify, while transducing the activation signal, and decay at four different probabilities. Changes in whole-gene expression during human early embryogenesis and hematopoiesis are reproduced in simulation using this epigenetic learning process in a single genetically-determined hierarchical-pair architecture of gene regulatory cascades. On the background of this learning process, I propose the law of biological inertia, which means that a living cell basically maintains the expression pattern while renewing its contents.

## Introduction

A living cell is a complex adaptive system. The expression of a gene is controlled by many mechanisms, including transcription factors, chromatin modifications, and non-coding RNAs. Fine regulation of multiple genes is required for a cell to function appropriately, depending on the cell type and environment. Big omics data, including whole gene expression data in a single cell, are accumulated by using single-cell RNA sequencing (scRNA-seq) and other technologies. Based on these data, systems biology proposes gene regulatory networks (GRNs) that generate outputs from inputs^1–3^. However, the modeling of complicated GRNs for many genes requires tuning of numerous parameters. It remains unclear how the expression level of more than 10,000 genes is properly controlled in a cell^1^.

While molecular biology investigates causal relationships in cells as if they were well-designed machines, superior machines have acquired learning ability. Deep reinforcement learning and the AlphaGo algorithm in computer science have made great advances to play board games^4,5^. Prior to these technologies, conventional software could not overcome professional board game players, even after collecting large volumes of data and tuning many parameters^4^. Deep reinforcement learning includes a deep neural network and a Monte Carlo tree search. The neural network is a multilayer architecture, through which input data with high dimensionality are processed using a weight matrix^6^. Error is calculated as the difference between the output and the correct answer to alter the weight matrix through backpropagation. Monte Carlo tree search stochastically selects a series of actions^5^. By repeating these trial-and-error processes, the algorithm determines an optimal weight matrix for selecting correct actions in any situation. The learning processes may be required for cells in which there are too many situations to prepare in advance^7^.

The importance of stochastic and feedback processes is proposed in complex adaptive systems^7–9^. Waddington epigenetic landscape schematically visualizes the processes through which a cell autonomously, not deterministically, reaches an appropriate gene-expression pattern^10,11^. A cell may alter the gene expression depending on the inappropriateness of the current pattern. This is a kind of learning process. In this study, I theoretically show that biological processes in gene expression can regulate the expression of the whole gene at appropriate levels by acting as a learning process.

## Results

### Amplification and error-dependent decay for learning

**Figure 1:**
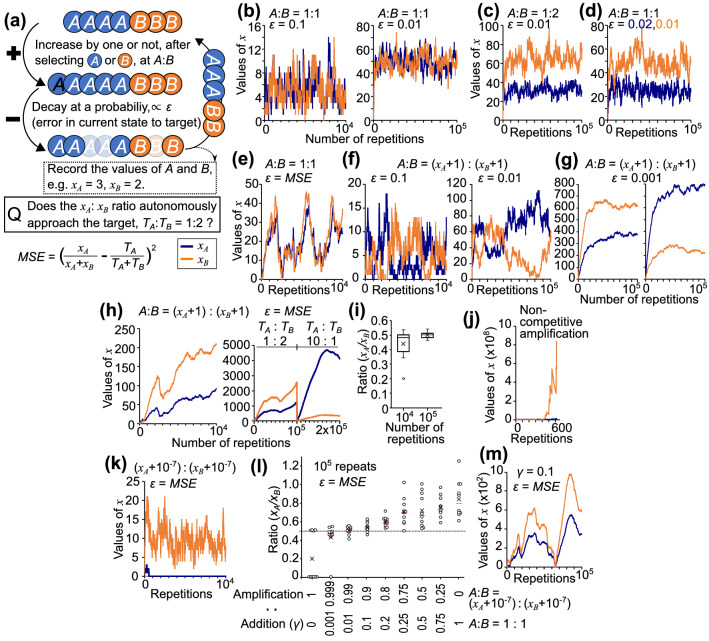
Regulation of two factors through stochastic processes of increase and decrease. (**a**) Scheme of the simulation to reveal minimum processes required for learning. The non-negative integer values of factors *A* and *B*, *x*_*A*_ and *x*_*B*_, change by repeating stochastic processes of increase and decrease. For every 10 repeats on average, *A* or *B* is selected at an *A*:*B* ratio, increasing the selected *x*_*A*_ or *x*_*B*_ by one unit. Each unit in *x*_*A*_ and *x*_*B*_ disappears at a probability of 0.1*ε*, due to the decay every repeat. See text for concrete examples, Table 1 for the definition of parameters, and Fig. 6 for the mathematical formula. (**b**–**m**) The number of repetitions of these processes are indicated on the x-axis (**b**–**k**, **m**). The values of *x*_*A*_ (blue) and *x*_*B*_ (orange) (**b**–**h**, **j**, **k**, **m**) and the *x*_*A*_/*x*_*B*_ ratio (**i**, **l**) are shown. (**b**) Additive increase at a 1:1 ratio and decay with a constant probability. (**c**) Additive increase at a 1:2 ratio and decay with a constant probability. (**d**) Additive increase at a 1:1 ratio, and *x*_*A*_ and *x*_*B*_ decay with constant probabilities of 0.002 and 0.001, respectively. (**e**) Additive increase at a 1:1 ratio and decay with probability equal to the *MSE* between the current (*x*_*A*_:*x*_*B*_) and target (1:2) ratios. (**f**, **g**) Competitive amplification with a bias term (increase by one, selecting *A* or *B* at a (*x*_*A*_ + 1):(*x*_*B*_ + 1) ratio), and decay with a constant probability. (**h**, **i**) Competitive amplification and *MSE*-dependent decay. Target ratio *T*_*A*_:*T*_*B*_ is changed from 1:2 to 10:1 after 10^5^ repeats. (**i**) Ratios of *x*_*A*_ to *x*_*B*_ in 10 tests are shown in box and whisker plots indicating the interquartile range, 1.5 × the interquartile range, the mean (cross), and the data in outlier region (circle). (**j**) Non-competitive amplification and *MSE*-dependent decay. (**k**) Competitive amplification without additive increase setting bias *β* = 10^−7^, and *MSE*-dependent decay. (**l**, **m**) Competitive amplification without bias (*A*:*B* = (*x*_*A*_ + 10^−7^): (*x*_*B*_ + 10^−7^)) or additive increase (*A*:*B* = 1:1) is selected at a (1 − *γ*):*γ* ratio in an increase process. The *x*_*A*_/*x*_*B*_ ratio at 10^5^ repeats in 10 tests are shown in circles with the mean (cross) and median (red bar) in (**m**). The target ratio 0.5 is indicated by a dotted line.

I attempt to clarify the processes through which factors autonomously reach their target ratio without individual commands by using a simple simulation model with two factors (Fig. 1a; Table 1). Here, the non-negative integer values of two factors, *x*_*A*_ and *x*_*B*_, change by 10^4^–10^5^ repeats of stochastic processes of increase and decrease from 1, which is set as the initial value. The target ratio, *T*_*A*_:*T*_*B*_, is set to 1:2. In the increase process, which proceeds at a probability of *α*_*inc*_ = 0.1, either *A* or *B* is selected, and the value of the selected factor increases by one. *x*_*A*_ and *x*_*B*_ stochastically decay at a probability *α*_*dec*_* ε*, where *α*_*dec*_ = 0.1. Thus, after a decrease process, *x*_*A*_ decays to a value selected from a binomial distribution with the number of trials *x*_*A*_ and the probability (1 − 0.1 *ε*). In this text, the assumption or settings are written in the present tense, whereas the results of simulation are written in the past tense.

**Table 1:** Variables and parameters in the models.

Indicator	Meaning	Values	Comments
*A, B*	Identifier of two factors	*A*:*B* indicates the ratio of selection in increase process	
*x*_*A*_, *x*_*B*_	Value of each factor	Non-negative integer variables	Changing by increase and decrease
*T*_*A*_:*T*_*B*_	Target ratio of each factor	1:2 (Fig. 1, except for Fig. 1h after 10^5^ repeats)	Calculated from RNAseq data (Figs. 2k, 3, 4, 5)
*α*_*inc*_	Probability to enter increase process at each repetition	Constant value 0.1 in Figs. 1, 2	*α*_*inc*_ is replaced by the Monte Carlo tree search in the model with an mRNA in Figs. 4 and 5
		Variable (0.01–0.101) depending on the coverage of the pair in the whole in Fig. 3c–f	
*α*_*dec*_	Constant coefficient of decay probability	0.1	Applied in Figs. 1 and 2
	Probability to enter decrease process at each repetition		Applied in Figs. 3, 4 and 5
*β*_*A*_*, β*_*B*_	Bias. White noise, when *β*_*A*_ = *β*_*B*_. In amplification, select A or B at a (*x*_*A*_ + *β*_*A*_):(*x*_*B*_ + *β*_*B*_) ratio	1 in Figs. 1f–i, 2–3, 4a–f, while 10^−7^ in Figs. 1k–m, 4g–i	Constant value to increase additively and to avoid extinction in amplification
*MSE*	Mean squared error between current and target ratios	$\left( {x_{A} /\left( {x_{A} + x_{B} } \right) - T_{A} /\left( {T_{A} + T_{B} } \right)} \right)^{2}$	$= \left( {x_{B} /\left( {x_{A} + x_{B} } \right) - T_{B} /\left( {T_{A} + T_{B} } \right)} \right)^{2}$ Same value for *A* and *B*
*ε*	Error, which is equivalent to a parameter of decay probability	Constant (Fig. 1b–d, f, g)	*x*_*A*_ values after a decrease process is randomly selected from binomial distribution with the number of trials *x*_*A*_ and the probability (1 − *α*_*dec*_* ε*) in Figs. 1 and 2 or (1 − *ε*_*(x)*_) in Figs. 3, 4 and 5 4-step error is applied in Fig. 5
		*MSE* (Figs. 1e, h–m, 2b, c, i)	
		*MSE* in Figs. 2, 3, 4 and 5 is rounded to 10^−1^, 10^−2^, 10^−3^, …, 10^−*i*^ in stepwise, to 10^−1^, 10^−2^, 10^−3^ in 3-step error, and to 10^−1^, 10^−2^, 10^−3^, 10^−4^ in 4-step error	
*γ*	Probability to choose additive increase among increase processes	0 or an indicated constant in range from 0 to 1	This *γ* is used only in Fig. 1l, m
			0 in other figures
Initial ratio	Ratio of each factor in the total at the initial setting	Even distribution in Figs. 1 and 2	
		Expression ratio of genes from RNA-seq data in Figs. 3, 4 and 5	
Initial value of a branch in a pair		1 in Figs. 1 and 2	
		Initial ratios are summed for genes in the branch of the pair, multiplied by the number of genes (11,281), and rounded to make an integer value, in Fig. 5	

In the learning pair model, the values of two factors, *x*_*A*_ and *x*_*B*_, repeat stochastic processes of increase and decrease. In the increase process, either *A* or *B* is selected, and the value of the selected factor increases by one. In the increase process, competitive amplification or additive increase is chosen at (1 − *γ*):*γ* ratio. In competitive amplification, *A* or *B* is selected at the (*x*_*A*_ + *β*_*A*_):(*x*_*B*_ + *β*_*B*_) ratio. In the additive increase, *A* or *B* is selected at a 1:1 ratio. In the decrease process, *x* decreases by decay at a probability depending on the error value *ε.*

For a concrete example, two factors *A* and *B* denote family genes *A* and *B* that have high similarity in the promoter regions. The value *x*_*A*_ indicates the amount of acetylation of histones, or the openness degree of chromatin, at the gene *A* locus. Then, the ratio of *x*_*A*_:*x*_*B*_ is equivalent to the mRNA amount ratio. The delay time from the chromatin modification to the change of protein amounts is not implemented in this simulation. In other possible interpretations, *x*_*A*_ and *x*_*B*_ denote the number of type *A* cells and *B* cells in a tissue, the cross-sectional area of main and side branches of a living tree, or the degrees of synapse connectivity of a bifurcating neuron. This abstract model can be applied to various biological phenomena in which two related individual-things are autonomously controlled to an appropriate ratio.

In the first model, *x*_*A*_ and *x*_*B*_ are assumed to change at a fixed probability (Fig. 1b–d). Either *x*_*A*_ or *x*_*B*_ is selected at a 1:1 ratio for the increase, and the decay probability is fixed at *ε* = 0.1 or 0.01. Simulation results showed similar values in *x*_*A*_ and *x*_*B*_ (Fig. 1b). If the probability of increase in *x*_*B*_ is two-fold of that in *x*_*A*_ (Fig. 1c) or if the decay probability in *x*_*A*_ is two-fold of that in *x*_*B*_ (Fig. 1d), the *x*_*A*_/*x*_*B*_ ratio approached the target ratio, 0.5. However, these conventional models with individualized probabilities require something that determines the appropriate parameter-setting.

In the second model (Fig. 1e), the decay probability is the same for *x*_*A*_ and *x*_*B*_ but changes over time, taking a value that is the mean squared error (*MSE*) between the current and target ratios: $\varepsilon_{\left( x \right)} = MSE = \left( {x_{A} /\left( {x_{A} + x_{B} } \right) - T_{A} /\left( {T_{A} + T_{B} } \right)} \right)^{2}$. Regarding the increase, either *x*_*A*_ or *x*_*B*_ is selected at a 1:1 ratio. The dynamics of *x*_*A*_ and *x*_*B*_ exhibited a pattern similar to predator–prey in ecology, in which the fluctuation in the number of prey *x*_*B*_ slightly preceded that of predator *x*_*A*_.

In the third model (Fig. 1f, g), *x*_*A*_ and *x*_*B*_ are assumed to increase by competitive amplification, in which either *x*_*A*_ or *x*_*B*_ is selected at a ratio of (*x*_*A*_ + *β*_*A*_):(*x*_*B*_ + *β*_*B*_) to increase by one, where bias *β*_*A*_= *β*_*B*_= 1. When the decay probability *ε* = 0.1 or 0.01, *x*_*A*_ and *x*_*B*_ fluctuated with a switching pattern in which either *A* or *B* dominated transiently (Fig. 1f). When *ε* is as low as 0.001, the *x*_*A*_/*x*_*B*_ ratio persisted at a certain value that was stochastically determined at early time points (Fig. 1g).

In the fourth model (Fig. 1h, i), *x*_*A*_ and *x*_*B*_ are assumed to increase by competitive amplification as in the third model, and to decrease by decay with a probability of *MSE* between the current and target ratios as in the second model. The simulation results showed that the *x*_*A*_/*x*_*B*_ ratio approached the target ratio of 0.5. Some deviations observed at 10^4^ repeats were reduced after 10^5^ repeats of stochastic processes (Fig. 1i). This model was applicable for other target ratios (Fig. 1h) without tuning parameters. Thus, repeating stochastic processes of competitive amplification and *MSE*-dependent decay is a system that autonomously learns the target ratio through trial-and-error. The epigenomic regulation of chromatin modification can be interpreted as a competitive amplification process (Table 2)^12^. Importantly, actual cells do not know the final target ratio (the correct mRNA ratio) a priori while approaching the target with *MSE*-dependent decay. As an example of the decay, high stress due to improper expression reduces transcription rates via RNA-mediated epigenomic modification^13^.

**Table 2:** Assumptions in the learning hierarchical-pair model are supported by biological knowledge.

Model assumptions	Biological findings	Regulation
Competition	A transcription factor chooses a binding locus among candidates, depending on the openness ratio of the chromatin	Epigenomic
Amplification	Transcriptional coactivators with histone acetyltransferase activity relax the chromatin structure	
	Transcription opens the chromatin, and the open chromatin structure induces transcription	
Bias (no extinction) Additive increase	Whole-genome in every somatic cell	Genetic
	Conventional genetic regulation of transcription	
Error (approximated)-dependent decay	Cellular stress responses	Dependent on cell and environment Feedback from the current fitness
	Rough evaluation of the current state	
	Histone deacetylases and DNA methyltransferases close the chromatin structure	
	Non-coding RNA-dependent cleavage	
	RNA-mediated epigenomic modification	
Hierarchical-pair architecture	Signal transduction cascades for gene expression	Genetic
	Topologically associated domains (TADs)	
Competitive amplification in hierarchical pairs	Active and expressed cascades are preferentially selected and activated	Cell-type dependent Post-translational
	Kinase is activated by phosphorylation at multiple sites	
Error-dependent decay in hierarchical pairs﻿	Cellular stress responses	Dependent on cell and environment
	Dephosphorylation	
	Polyubiquitin dependent degradation	
	RNA-mediated epigenomic modification	

Epigenetic regulations, which are highly variable depending on cell type, can be interpreted as a process of competitive amplification. The decay rate is roughly regulated at several levels by the fitness of the current expression pattern in each pair. The correct expression level of each gene is not supervised in real cells. Instead, two functionally related gene-groups are regulated in a pair, in which the inappropriate expression ratio induces cellular stress, increases the decay, and destabilizes the ratio. As a possible feedback regulation for the error-dependent decay, cleaved mRNA fragments coding excessive proteins may close the genome loci. Hierarchical pairs are genetically determined and consistent in all cell-types.

Amplification may induce a large difference in the value of each factor by exponential growth, making a factor all or nothing. In non-competitive amplification, in which either *A* or *B* is selected at a 1:1 ratio and the selected term increases by *x*_*A*_ + 1 or *x*_*B*_ + 1, *x*_*B*_ reached a much higher value than *x*_*A*_ (Fig. 1j). When bias *β* in competitive amplification is not 1 but rather 10^−7^ (which is almost equivalent to 0 and avoids the 0/0 error in processing), *x*_*A*_ decreased to 0 in six of the ten tests (Fig. 1k, l left). Interestingly, the *x*_*A*_/*x*_*B*_ ratio approached the target ratio in the other four tests. Competition and the addition term of bias are required to avoid extinction in amplification.

In our previously reported immune response model, three processes were assumed to occur during changes in the interaction intensity or cell number: competitive amplification (proliferation), regulated reduction (dissociation), and additive increase (migration)^14^. Based on the model, a process of additive increase, in which either *x*_*A*_ or *x*_*B*_ is selected at a 1:1 ratio to increases by one, is chosen at a probability *γ* in an increase process (Fig. 1l). The condition *γ* = 0 is equivalent to that in Fig. 1k, whereas *γ* = 1 is equivalent to that in Fig. 1e. As *γ* is set to a lower value, the *x*_*A*_/*x*_*B*_ ratio after 10^5^ repeats became skewed from 1 to 0.5 (target ratio). When *γ* is negligibly low, *x*_*A*_ sometimes disappeared. When the additive increase is chosen at low probabilities (*γ* = 0.01, 0.1), the *x*_*A*_/*x*_*B*_ ratio approached the target ratio (Fig. 1l, m). Therefore, the role of bias term (*β* = 1) is equivalent to this small additive increase or white noise.

The learning process can be explained as follows. When *MSE* and decay probability are high, the *x*_*A*_/*x*_*B*_ ratio fluctuates in full range, like a rough adjustment, by avoiding the extinction using bias (additive increase) or noise (Fig. 1f). The *x*_*A*_/*x*_*B*_ ratio is improved on average by the error-dependent decay, which is a random walk with smaller step-size as it gets closer to the target. When the *x*_*A*_/*x*_*B*_ ratio approaches the target ratio, the ratio persists because the decay rate becomes low and because *A* or *B* becomes selected with the ideal ratio. The changes in this autonomously-reached stable state are equivalent to those in conventional models (Fig. 1c), in which the parameters need to be set accurately in advance in contrast to the learning model. In the main simulation hereafter, competitive amplification implies selecting *A* or *B* at a (*x*_*A*_ + 1):(*x*_*B*_ + 1) ratio to increase by one with *β* = 1, *γ* = 0. This is designated as a learning pair process.

### Hierarchical pairs and approximated *MSE*

**Figure 2:**
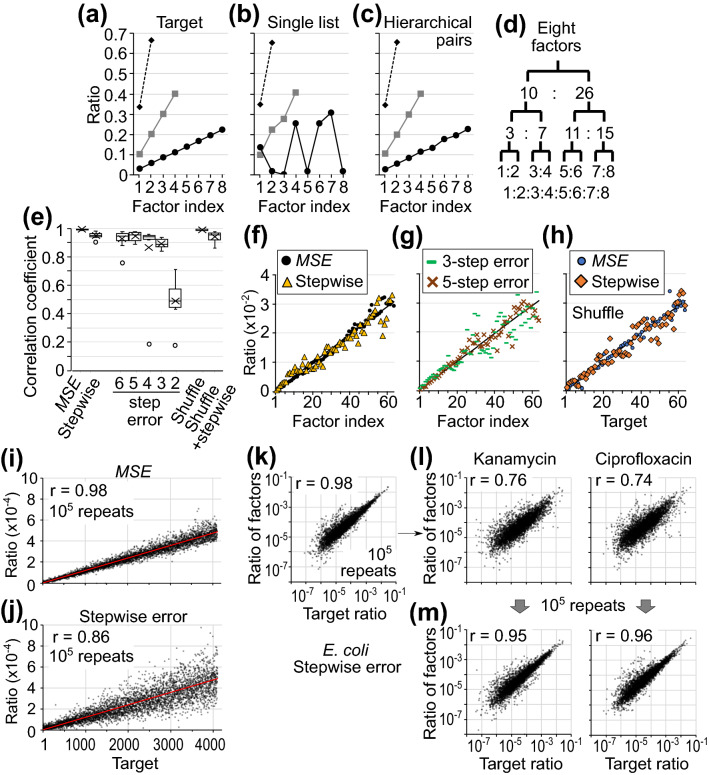
Regulation of multiple factors by using hierarchical pairs and approximated error. (**a**–**d**) Stochastic processes of competitive amplification with *β* = 1 and *MSE*-dependent decay are repeated in the model with two, four, and eight factors. (**a**) Target ratios of each factor in three simulation conditions are shown as three lines with markers. (**b**) Competition and *MSE* are calculated in one list that include all factors. The ratios of each factor after 10^6^ repeats are shown. (**c**, **d**) The ratios of eight or four factors are determined by hierarchical pairs (**d**). Each pair independently repeats the stochastic processes 10^5^ times. (**e**–**h**) The results after 10^5^ repeats in the model regulating 64 factors in hierarchical pairs. In *MSE*, the error is calculated with full accuracy. In “stepwise”, *MSE* is rounded every 10 folds. The number of steps of error (**g**) indicates the possible error levels. In “shuffle” (**h**), the factor with each target value is randomly set in the hierarchical pairs. In (**e**), correlation coefficient between the target and result ratios in 10 tests are shown in box and whisker plots indicating the interquartile range, 1.5 × the interquartile range, the mean (cross), and the data in outlier region (circle). Black lines in (**f**–**h**) indicate the target ratios. (**i**–**m**) The results in the model with 2^12^ factors after 10^5^ repeats. Accurate *MSE* (**i**) or approximated stepwise error (**j**–**m**) is applied to decay probability. (**i**, **j**) The factor with each target value (range 1–4096 as indicated by a red line) is randomly set in the hierarchical pairs. (**k**) The target ratio is set using the expression ratio in *E. coli* without antibiotics. Initial condition of pairs is an even distribution. (**l**, **m**) Subsequently, from the (**k**) state, the target ratios for the next 10^5^ repeats are reset using gene expression data in the presence of antibiotics. The ratios of the factors before (**l**) and after (**m**) the second 10^5^ repeats are shown, where r is the correlation coefficient.

To regulate gene expression, more than two factors must be controlled. When the ratios of four or eight factors are examined to be controlled by competitive amplification and *MSE*-dependent decay, the value ratios of eight factors in a single list failed to approach the target ratios (Fig. 2a, b). The eight factors can be divided into seven pairs in three layers (Fig. 2c, d). The fraction of each factor in total is calculated as an infinite product of all ratios in the pairs that include the factor. When the values in each pair independently change by the stochastic learning pair process, eight factors successfully approached the target ratio after 10^5^ repeats (Fig. 2c).

Next, the required accuracy of *MSE* is tested because accurate detection of errors is difficult in vivo. When the *MSE* between the current and target ratios is accurately calculated, 64 factors approached the target ratio, which is set as a linear distribution in the range of 1–64 (correlation coefficient between the target and result ratios after 10^5^ repeats, r, was 0.99, Fig. 2e, f). As an approximation of *MSE*, the calculated *MSE* is rounded to 10^−1^, 10^−2^, 10^−3^, … ,10^−*i*^, where *i* is a natural integer, in stepwise error. When this approximated error is used, the correlation between the result and target ratios decreased but remained high (r = 0.95, Fig. 2e, f). By setting a maximum value for *i* that indicates the lower limit of the stepwise error, five additional types of approximated *MSE* are compared (6-, 5-, 4-, 3-, and 2-step error in Fig. 2e, g). The results indicated that 3-step error was required for learning (median r = 0.89) and that the stepwise error was almost equivalent to 5-step error (median r = 0.95). The approximation of *MSE* decreased the learning accuracy, but multiple factors in the model using stepwise detection of error approached the target ratio to an acceptable level.

Next, the relationship between indexes for making pairs and targets is randomly shuffled. The ratios of each factor after 10^5^ repeats approached the target ratios to an almost equivalent level to that without shuffling (Fig. 2e, h). Furthermore, 4096 = 2^12^ factors approached the target that is set to shuffled values ranging from 1 to 4096 (r = 0.97–0.98 with accurate *MSE* and r = 0.84–0.91 with stepwise error in five tests, Fig. 2i, j).

When the gene expression data from bacteria without antibiotics (GSM2538622 RNA-seq dataset)^15^ are used as the target ratio, the ratio of 4096 factors changed from the initial even-distribution to the expression pattern after 10^5^ repeats of stochastic processes with stepwise error (r = 0.98, Fig. 2k). Subsequently, when the target ratios are reset to the gene expression pattern observed in the presence of antibiotics (Fig. 2l)^15^, the ratio of 4096 factors changed from the pattern without antibiotics to the new target pattern (Fig. 2m). Thus, bacteria may autonomously produce proper gene expression patterns by reducing the error caused by antibiotics.

### Hierarchical clustering of human genes

**Figure 3:**
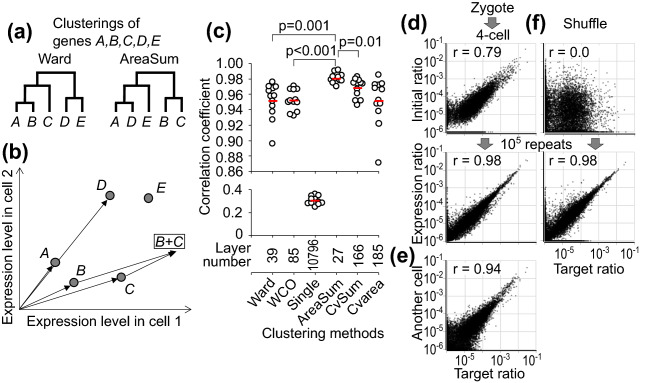
Hierarchical clustering of genes for the learning pair model. (**a**, **b**) Dendrograms (**a**) are generated using hierarchical clustering methods from the gene expression pattern (**b**). (**c**) Using the expression of 16,921 genes in 20 cells from human early embryos, 6 clustering methods generated hierarchical pairs with the indicated number of layers. In 12 tests using 3 zygote data for initial setting and 12 4-cell data for target ratio, each pair in hierarchical pairs changes the values for 10^5^ repeats in the learning pair model with stepwise error. The correlations to the target ratio are shown with the mean (red bar). Paired *t*-test is used for statistical analysis. (**d**) Using hierarchical pairs generated with the AreaSum method and stepwise error, the change from zygote to 4-cell stage is tested. Relative expression ratios of 16,921 genes before and after 10^5^ repeats are plotted against the target ratio. (**e**) Gene expression ratios from another dataset of the 4-cell stage are plotted against the target ratio using published scRNA-seq data^19^. (**f**) In the same settings in (**d**), the initial and target ratios are independently shuffled. In the dot plots, relative ratios are plotted after adding 10^−6^ to all genes.

I next apply the learning hierarchical-pair process to human gene expression. In advance, it is necessary to set the genes that are paired. Six hierarchical clustering analysis methods (Fig. 3a–c), which are Ward, WCO, Single, and three newly-developed methods (AreaSum, CvSum and Cvarea), are applied to a total of 16,921 genes in 20 differently labeled cells from preimplantation human embryos, human embryonic stem cells, and downstream early mesoderm and endoderm progenitors (scRNA-seq datasets E-MTAB-3929, GSM2257302, and GSE75748)^16–18^. The number of layers in hierarchical pairs generated by the AreaSum method was the smallest (27), whereas that by the Single method was largest (10,796) (Fig. 3c). In the AreaSum method, the area formed by two vectors from the origin is calculated as the distance between two genes, and the total gene expression level is used as the representative value of the cluster (Fig. 3a, b).

As another modification, the probability of entering a process of competitive amplification, *α*_*inc*_, is set as a variable in the range of 0.001–0.101 depending on the coverage of the pair. This assumption is further modified in another model with an mRNA pool. For test data, another scRNA-seq dataset from human preimplantation embryos (GSE36552) is used^19^. The initial and target ratios in each pair are set with the data of a zygote and a cell at 4-cell stage, respectively. The correlation coefficient between the initial and target ratios is a median r = 0.78 (range 0.67–0.84) in 12 tests (Fig. 3d). For each pair, the stochastic processes of competitive amplification and decay using the stepwise-approximated *MSE* are repeated 10^5^ times.

The learning efficiency was compared among the six different hierarchical-pair architectures. The expression ratio most closely approached the target ratio when hierarchical pairs generated by the AreaSum method are used (r = 0.98, Fig. 3c, d), with even a closer correlation than another 4-cell data in scRNA-seq (Fig. 3e)^19^. In pairs generated by the Single method, the expression ratio did not approach the target ratio (Fig. 3c). These results indicate that the architecture of hierarchical pairs affects the ability to approach the target ratio. In contrast, even when the initial and target patterns are independently shuffled to test non-correlated artificial patterns, the expression ratio approached the target ratio (median r = 0.98, range 0.94–0.99 in six tests) in the hierarchical pairs generated by the AreaSum method (Fig. 3f). Owing to the high adaptability of this learning process, it was difficult to validate the accuracy of gene pairing.

### A model with a signal transduction cascade and an mRNA pool

**Figure 4:**
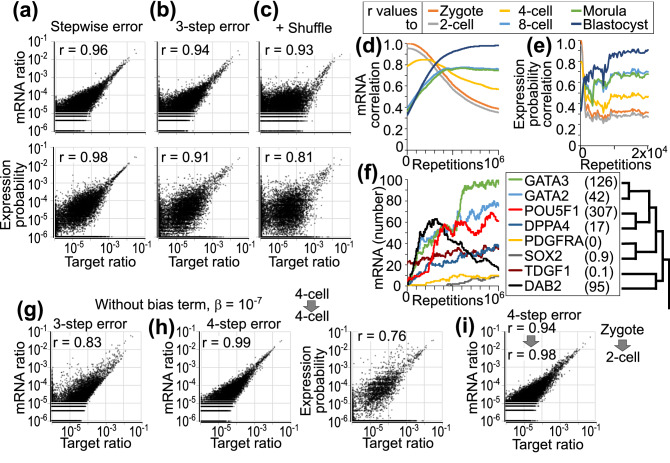
Model with an mRNA pool. In the model with an mRNA pool and hierarchical pairs generated by the AreaSum method, stochastic processes are repeated for 5 × 10^5^ times. The expression probability is equivalent to the expression ratio in Fig. 3d. (**a**) The change from zygote to 4-cell stage with stepwise error. (**b**–**g**) Model with 3-step error. (**b**) The change from zygote to 4-cell stage is tested. (**c**) Initial and target ratios are set with independently shuffled zygote and 4-cell data. Ratios after 10^6^ repeats are shown. (**d**–**f**) Initial and target ratios are set with zygote and blastocyst. Simulation data of mRNA and expression probability are recorded every 250 repeats. (**d**) Correlation coefficient between the mRNA ratio and the indicated scRNA-seq data. (**e**) Correlation coefficient between the expression probability and scRNA-seq data are plotted until 2 × 10^4^ repeats. (**f**) The amount of indicated genes in the mRNA pool is plotted. The numbers in parentheses indicate the target level of each gene. The dendrogram indicates the layers in which the genes are paired in the hierarchical pairs. (**g**–**i**) Bias *β* is set 10^−7^, not 1, for the homeostatic state. (**g**) Model with 3-step error. Initial and target ratios are set with the same 4-cell data. (**h**, **i**) Model with 4-step error. (**h**) Initial and target ratios are set with the 4-cell data. (**i**) Initial and target ratios are set with zygote and 2-cell stage. In the dot plots, relative ratios are plotted after adding 10^−6^ to all genes.

I assume that the hierarchical-pair architecture is a signal transduction cascade to select a gene for transcription in a model with an mRNA pool. Rather than using parameter *α*_*inc*_, a pair is stochastically selected at each repetition among pairs in the top seven layers depending on the coverage of the pair. In the selected pair, the competitive amplification is performed; branch *A* or *B* is selected at a ratio (*x*_*A*_ + *β*):(*x*_*B*_ + *β*), where *β* = 1, and the value of the selected branch, *x*_*A*_ or *x*_*B*_, increases by one. Additionally, the downstream pair of the selected branch enters the process of competitive amplification until the selected branch is a leaf indicating a single gene. In an mRNA pool, mRNA of the selected gene increases by one, with randomly replacing one mRNA. Initially, 360,000 mRNAs in the mRNA pool are set based on the initial ratio (zygote). In addition to the mRNA, the expression probability is calculated as an infinite product of ratios in pairs including the gene, which is equivalent to the expression ratio in the previous model without an mRNA pool. The ratios of mRNA and expression-probability approached the target ratio (4-cell) after 5 × 10^5^ repeats (r = 0.95–0.97 and r = 0.97–0.99, respectively, in six tests), although genes with 0 to several mRNAs were plotted discretely in mRNA ratios (Fig. 4a). Furthermore, even when decay probability or *MSE* is approximated to three different values, 0.1, 0.01, and 0.001 (3-step error as in Fig. 2e), similar changes approaching the target ratio were observed with setting 4-cell as the targets (r = 0.94–0.98 in 12 tests, Fig. 4b) and with shuffling the targets (r = 0.92–0.94 in six tests, Fig. 4c).

Next, to analyze the dynamics in the simulation, the initial and target ratios are set with zygote and blastocyst data. The mRNA ratio gradually approached the target blastocyst pattern over 10^6^ repeats, but not via the patterns of the 2-cell, 8-cell, or morula stages (Fig. 4d). The expression probability more quickly and directly reached near the target ratio within 10^4^ repeats (Fig. 4e), and then the similar correlation levels persisted during 10^4^–10^6^ repeats. The mRNA levels of each gene approached the target level with fluctuations (Fig. 4f). In a simulation, the dynamics of *GATA3* were more similar to those of *GATA2* than those of *DAB2*, although the initial and target values of *GATA3* and *DAB2* are closer than those of *GATA2*. The higher correlation during stochastic fluctuations is explained by the hierarchical-pair architecture, where *GATA2* and *GATA3* are paired in the 7th layer from the top, whereas they are separated from *DAB2* in the 2nd layer.

For gene regulation during homeostatic state, the bias term *β* may not be required because *MSE* or decay rate can be kept low. When both the initial and target ratios are set with the same 4-cell data, the mRNA ratio deviated from the pattern during 5 × 10^5^ repeats in the model with *β* = 10^−7^ and 3-step error (r = 0.66–0.88 in six tests, Fig. 4g). In contrast, the mRNA ratio maintained the set pattern in the model with 4-step error at least for 5 × 10^5^ repeats (r = 0.98–0.99 in six tests), while the correlation between expression probability and the target ratio gradually decreased (r = 0.76–0.87, Fig. 4h). When the initial state is set with a zygote and the target ratio is set with scRNA-seq data of 2-cell stage, which has a highly-similar expression pattern to a zygote (r = 0.94–0.96)^19^, the mRNA ratios approached the target ratio, except for one case in six tests (median r = 0.98, range 0.51–0.98, Fig. 4i). However, the change from a zygote to the 4-cell stage was poorly reproducible in the model with *β* = 10^−7^ (median r = 0.88, range 0.85–0.94 in six tests). In the absence of the bias term or white noise, a homeostatic state with a similar expression pattern was maintained while allowing some limited changes in differentiation.

### A common model for human gene expression

Based on these findings, I propose that a single model can control whole gene expression during any differentiation processes in human cells and evaluate this in early embryogenesis and hematopoiesis. To generate hierarchical pairs, I collect 13 scRNA-seq datasets from human tissues^16–18,20–29^, in which 11,281 gene names were commonly labeled in 11,803 cells. Using the relative expression ratio of these 11,281 genes in each cell, a hierarchical-pair architecture was generated using the AreaSum clustering method. This architecture contained 11,280 pairs in 22 layers (Supplementary Table 1).

**Figure 5:**
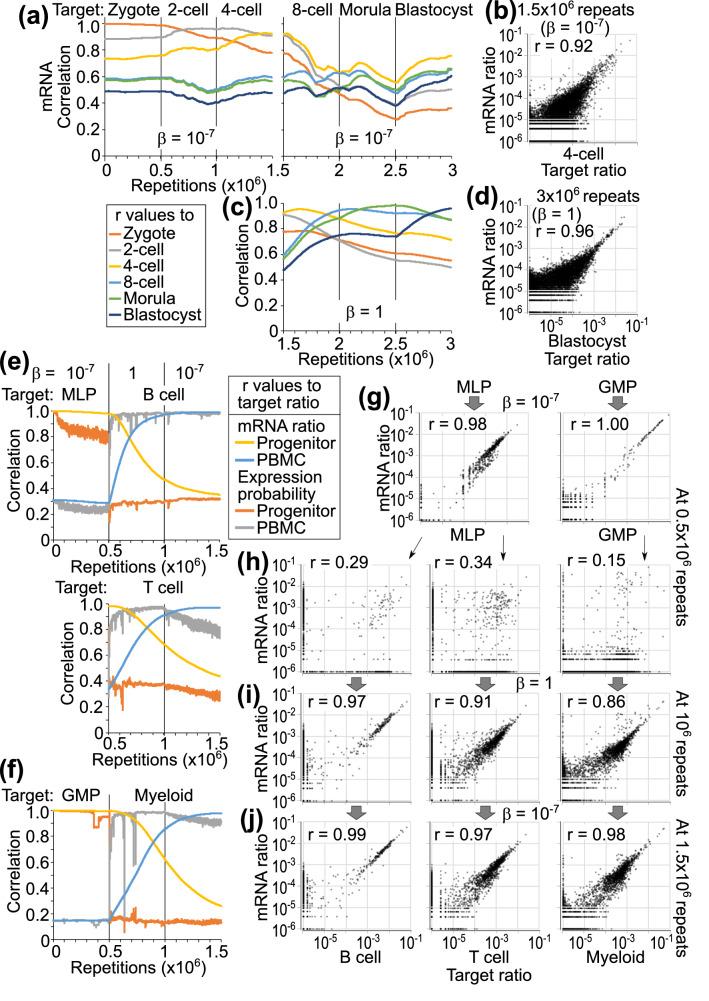
Single model of whole gene expression in early embryogenesis and hematopoiesis. (**a**–**d**) Learning hierarchical-pair model with 11,281 genes, an mRNA pool, and 4-step error is applied to differentiation from human zygote to blastocyst. (**a**) The initial ratio is set with zygote data. Target ratio is changed every 5 × 10^5^ repeats from zygote to 2-cell, 4-cell, 8-cell, morula, and blastocyst stages. Bias *β* is set 10^−7^. Correlation coefficient between the ratios of mRNA and each target are calculated every 250 repeats. (**b**) Ratios of 11,281 genes in mRNA pool after 1.5 × 10^6^ repeats are plotted against the target 4-cell data. (**c**) Bias *β* is changed to 1 after 1.5 × 10^6^ repeats in (**a**). Target ratio is changed from 8-cell to morula and blastocyst stages every 5 × 10^5^ repeats. (**d**) Ratios of mRNA at 3 × 10^6^ repeats in (**c**) are plotted against the target blastocyst data. (**e**–**j**) The same model is applied to hematopoietic differentiation from progenitors (MLP and GMP) to PBMCs (B cell, T cell, and myeloid cell). Initial ratio is set with a progenitor. During the first 5 × 10^5^ repeats, bias *β* is set 10^−7^ and the target ratio is set with the same progenitor. During the next 5 × 10^5^ repeats, bias *β* is set 1 and the target ratio is changed to a PBMC. During the last 5 × 10^5^ repeats, bias *β* is set 10^−7^, keeping the same PBMC target. In the dot plots, relative ratios are plotted after adding 10^−6^ to all genes.

The model with an mRNA pool, 4-step approximated error, and this hierarchical-pair architecture is applied to the regulation of 11,281 genes, setting bias *β* = 10^−7^ or 1 depending on the situation. When the initial state of pairs and mRNA pool is set with a zygote scRNA-seq data, and the target ratio is changed in the order of zygote, 2-cell, 4-cell, 8-cell, morula, and blastocyst stages every 5 × 10^5^ repeats, the ratio in the mRNA pool dynamically approached the target ratios until the 4-cell stage in the model with *β* = 10^−7^ (Fig. 5a, b). When the model with *β* = 1 is applied after 1.5 × 10^6^ repeats, the gene expression patterns sequentially approached the 4-cell, 8-cell, morula, and blastocyst patterns with a correlation coefficient of more than 0.95 at the peaks (Fig. 5c, d).

In hematopoiesis, multi-lymphoid progenitors (MLPs) differentiate into B cells or T cells in peripheral blood mononuclear cells (PBMCs), whereas granulocyte–macrophage progenitors (GMPs) differentiate into myeloid cells^30^. When the initial state and target ratio are set with a progenitor, the mRNA ratios were maintained during 5 × 10^5^ repeats in the model with *β* = 10^−7^ (r = 0.97–1.0 in six tests, Fig. 5e–g). The expression patterns in progenitors are largely different from those in PBMCs^31^ (median r = 0.29, range 0.15–0.57 in 9 tests, Fig. 5h). When the target ratio is changed to a PBMC pattern, and *β* is set to 1, the mRNA ratio approached the target ratio during the next 5 × 10^5^ repeats (r = 0.86–0.97), with more rapid adaptation in the expression probability (Fig. 5e, f, i). The mRNA ratio, but not the expression probability, further approached the target ratio during the following 5 × 10^5^ repeats in the model with *β* = 10^−7^ (r = 0.97–0.99, Fig. 5e, f, j). These results demonstrate that the learning hierarchical-pair model using one common architecture can reproduce various differentiations and not-immortal homeostasis by adding bias terms in the former.

## Discussion

I propose a principle underlying whole gene regulation within cells, which includes learning ability and a common architecture of gene regulation. The learning ability is implemented as a repeat of two stochastic processes: competitive amplification in a pair and decay depending on *MSE* between the current and target ratios. The hierarchical structure of the pairs enables multiple factors to reach any target ratio.

**Figure 6:**
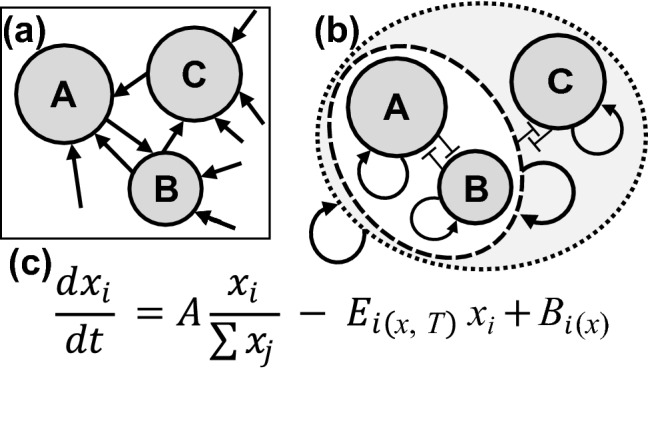
The law of biological inertia. (**a**) A conventional model of expression regulation of genes A, B and C. Each gene is regulated by others. The networks become complicated as the number of genes increases. (**b**) In the learning hierarchical-pair model, each gene or gene-cluster changes the expression level by amplification and decay, which are represented by self-regulation. Competition in each pair is presented as mutual inhibition. Two components in each dotted oval indicates a pair in a hierarchical architecture. The process in each pair proceeds in parallel. (**c**) The equation represents the law of biological inertia, which means that a living cell basically keeps the expression pattern while renewing the contents. The equation indicates that the *i*th gene transcription level changes by competitive amplification, error-dependent decay, and additive increase. In Fig. 1 in the range Σ*x*_*j*_ >> Σ*β*_*j*_, *x*_*i*_ $\in$ {*x*_*A*_, *x*_*B*_}, *A* = *α*_*inc*_(1 − *γ*), *E*_*i*(*x*, *T*)_ = *α*_*dec*_*ε*_*(x)*_*,* and *B*_*i(x)*_ = *γα*_*inc*_*β*_*i*_*/*Σ(*x*_*j*_ + *β*_*j*_). The openness degree of chromatin at the *i*th gene locus, *x*_*i*_, is maintained when *Ax*_*i*_/Σ*x*_*j*_ >> *B*_*i*_, where *B*_*i*_ is small noise, and *E*_*i*_ is the common lowest value.

In this model, the expression of each gene is regulated by itself, in contrast with conventional GRNs in which each gene is regulated by other genes (Fig. 6a, b). Conventional models require the control of all genes to appropriate expression levels in high-dimensional space. In contrast, in my model, the expression ratio of two functionally-related genes or gene clusters, such as GATA2 vs GATA3, actin-myosin vs microtubule, or mitochondria vs endoplasmic reticulum, is controlled by destabilizing the ratio when inappropriate. The simplicity of self-regulation in each pair is critical to increase the number of regulated genes in modeling, actual evolution, and organizing complex systems.

Importantly, the simple self-regulation system is not uncontrollable but rather efficient to generate a proper diversity. When *n* number of genes changes the expression at *L* levels, an infinitely large number of patterns *L*^*n*^ may exist. Conventional models set *m* number of master regulators (*m* << *n*) that control cell types and generate *L*^*m*^ states. The number of *L*^*m*^ is almost infinite and larger than the number of cell types we can understand, but it explains negligibly small space in *L*^*n*^. In my model, by using the four decay rates in *n* − 1 pairs, only 4(*n − *1) regulations are sufficient to generate any appropriate pattern. The increase probability of each factor in competitive amplification is autonomously tuned to the correct ratio, *x*_*i*_*/*Σ*x*_*j*_. Thus, amplification and stochasticity, which are misunderstood as interfering with strict control at a specific level, are essential for complex systems. I propose that the homeostasis, in which a cell keeps the expression pattern while the contents are metabolized, is not a result of complicated GRNs but a basic operating system shared in living things. This homeostatic system, which I refer to as the law of biological inertia, contains the learning process (Fig. 6c).

Biological knowledge of gene regulation is consistent with the assumptions in the learning hierarchical-pair model (Table 2). The first assumption, competition, is supported by the epigenomic regulation of transcription^12^. An RNA polymerase or transcription factor chooses a binding locus among candidates, depending on the local openness ratio of the chromatin. To be noticed, the binding candidates are genetically determined, as discussed in the next paragraph. This process would be repeated more than 10^5^ times during one cell-division cycle, estimated from the number of newly-generated mRNAs. The second assumption, amplification, is supported by positive feedback in the epigenomic regulation. The binding of transcription factors opens the chromatin at the locus, using cofactors with histone acetyltransferase activity. The third assumption, additive increase using a bias term, is supported by the fact that all somatic cells have the whole genome. The fourth assumption, decay rates dependent on the error between the current and target ratios, is not clearly described, possibly because the contribution is low under good cell-conditions. In speculation, when some proteins accumulate unused due to the inappropriate expression ratios, mRNAs for the excess proteins may be specifically cleaved, and the RNA fragments may close the chromatin. Histone deacetylase and DNA methyltransferase close the chromatin structure. Non-coding RNAs, which can be induced by biological stress, degrade a group of mRNAs with a particular sequence. RNA-mediated epigenomic regulations are described in several organisms^13^. Although many studies, especially for the regulation of decay in each pair, are required to demonstrate the molecular basis of the learning process, the assumptions are applicable to cells.

The hierarchical-pair architecture, the fifth assumption, is also supported by findings on topologically associating domains and signal transduction cascades for gene expression (Table 2). Topologically associating domains are conserved gene clusters with similar epigenomic states and high expression-correlations^32^. The learning pair process becomes more plausible in real cells by assuming that functionally related gene clusters are paired and regulated to the appropriate expression ratio. In the conventional view of signal transduction cascades, multimerization of specific receptors is assumed to deterministically trigger activation of a signal cascade to express a set of genes. However, the cascades and the induced genes vary depending on the cell type, which reflects the current expression and activation state. Active branches in the cascades may be preferentially used, just like the stochastic competitive amplification in the assumption. Further, many signal-transducing proteins are kinases that are activated by phosphorylation at multiple sites. Decay of activation is regulated by phosphatases and polyubiquitin ligases. The architecture of possible signal transduction, which is genetically determined by 3D molecular structure and promoter sequence, should be discriminated from the branch activity, which is regulated epigenetically or post-translationally (Table 2). In my model, the former genetic regulation is set as a common hierarchical-pair architecture conserved in all cells, whereas the latter epigenetic regulation is dynamically controlled following the basic law.

The learning hierarchical-pair model is consistent with the concept provoked by Waddington epigenetic landscape^10,11^ or the free-energy principle^8^, in which gene expression pattern becomes appropriate as if a cell rolls down on a landscape. Accordingly, the landscape itself or the gene-expression pattern under a new condition is not predictable in my model. Instead, the model predicts that cells are functionally robust to perturbations. A testable prediction is that the hierarchical architecture of regulated gene pairs is common in all human cells. Based on this single GRN, biologists will manipulate cells by predicting preferential differentiation or gene expression patterns.

My model differs from gene regulation in vivo in several aspects. First, the parameters are not based on experimental observations. Delay time for feedback regulation with *MSE* is ignored. Therefore, the time in the simulation does not linearly correlate with the actual time. Second, the bias *β* or additive noise should be controlled. Bias *β*_*A*_ and *β*_*B*_ may differ for each branch in each pair. Finely regulated bias is equivalent to an additive increase in gene or module activity, in which conventional deterministic regulations can be included. These additive bias terms are transiently and roughly required during differentiation, while the additive noise could be negligibly small or fixed to the correct values in terminally differentiated cells. Third, the calculation of the approximated error values from the target and current ratios is highly simplified. Pairs of crucial genes may be controlled more strictly, whereas many other pairs are controlled less strictly. Using scRNA-seq data as the target ratio, I show that gene expression reaches acceptable patterns for the cell. Fourth, the hierarchical pairs of genes generated by the AreaSum clustering method (Supplementary Table 1) should be revised to a true architecture. There is no evidence that my pairing is correct, because the shuffling of gene pairs did not significantly affect learning efficiency in the model with *β* = 1. Formally, forming a pair is equivalent to reducing the number of dimensions by one. The molecular biology of gene regulation, big data obtained by RNA-seq, and simulations and clustering using supercomputers would reveal the single correct gene-regulation architecture, which might be as useful as the periodic table of the elements in chemistry.

If the complexity of living organisms requires a template for increase, the increase of template would be formulated as competitive amplification. Death is formulated as error-dependent decay. A tissue composed of numerous cell-types regulates the cell ratio through proliferation (competitive amplification) and apoptosis (decay). In the immune system, we previously proposed that regulatory T cells, which are crucial for immune suppression, reduce decay probability and can be redefined as an indicator of low error^14^. Thus, the law of biological inertia provides insights for understanding various complex systems, implying the importance of individual freedom.

## Methods

### Computation

The simulation is performed using Python 3.7 software. Four files, including codes for the learning hierarchical-pair model (Code File 1), clustering of genes (Code File 2), model of human gene expression (Code File 3), and model with an mRNA pool (Code File 4), are available at GitHub. I perform Monte Carlo simulations in which the stochastic processes of increase and decrease are repeated 10^4^–10^6^ times for each pair as explained in Table 1 and Figs. 1a and 6. Note that, time does not indicate the actual time but the number of trials and errors (cycles of increase and decay). In the learning pair model, the values of factors *A* and *B* in each pair, *x*_*A*_ and *x*_*B*_, increase by one after selecting either *A* or *B* at an *A*:*B* = (*x*_*A*_ + *β*_*A*_):(*x*_*B*_ + *β*_*B*_) ratio as competitive amplification, where bias *β* is 1 if not indicated to be 10^−7^, and decays at *MSE*-dependent probability. In “shuffle”, the factor indexes for each target value and for the location in the hierarchical pairs are randomly shuffled to set randomized target values in the hierarchical pairs. The expression ratio of each factor in total is calculated as an infinite product of the ratios in all pairs containing the factor. In the text, the assumption or settings are written in the present tense, whereas the results of simulation are written in the past tense.

### Approximation of error

The *MSE* is calculated for each pair as the difference between the current and target ratios. In stepwise error, the value is expressed in exponential notation with a base of 10, the mantissa is rounded to 1, and only the exponent value is used as the level of error and as the decay probability. Accordingly, the stepwise error takes a value $\in \left\{ {10^{ - 1} , 10^{ - 2} , 10^{ - 3} , \ldots , 10^{ - i} , \ldots } \right\}$, where *i* is a natural integer. In 6-, 5-, 4-, 3-, and 2-step errors, the lower limit of the error value is set to 10^−6^, 10^−5^, 10^−4^, 10^−3^, and 10^−2^, respectively. Whereas the stepwise error may take an unlimitedly small value to zero, 6-step error can take six types of error, from 10^−1^ to 10^−6^, and 2-step error can take two types of error, 10^−1^ or 10^−2^. The code is available in Code File 1.

### Hierarchical clustering analysis

The hierarchical pairs in the learning hierarchical-pair model indicate groups of genes with similar expression patterns that might be controlled by a particular regulation-module. To generate optimal hierarchical pairs for the model, six hierarchical clustering analysis methods are compared.

Hierarchical clustering analysis repeats the following two calculations until a pair containing all genes is created: (1) pairing two genes or clusters with the closest distance, and (2) calculating the distances to the new cluster of genes. Ward method uses the Euclidean distance. The WCO method uses the cosine distance, which takes a high value in the case of a low correlation, and “weighted method” that is Weighted Pair Group Method with Arithmetic Mean (WPGMA). The Single method uses the Euclidian distance and “single method” that selects the nearest point in clusters. These three clustering methods are available in scipy.cluster.hierarchy.linkage of the Python tool. The three new clustering methods, AreaSum, CvSum, and Cvarea, are available in Code File 2. In these three methods, the total expression of genes in a cluster is used as the representative value of the cluster. This is appropriate because pairing in the learning hierarchical-pair model is equivalent to separation into two subgroups. In the AreaSum method, the area between two vectors from the origin to the values of the clusters is used as the distance. A small angle indicates a constant expression ratio among different cells. A large vector size allows genes with high expression to skip many layers in the hierarchical pairs. The two clusters with the smallest distance are paired. In the CvSum method, the total expression level of genes in the pair, including both branches, is summed for every cell, and the variation (cv) of the summed value among cells is used as the distance between two clusters. Family genes with functional substitutability can be paired. In the Cvarea method, the product of the area and the cv is used as the distance between two clusters.

In Fig. 3, hierarchical clustering analyses are applied to the expression of 16,921 genes in 20 cells. To generate the hierarchical-pair architecture used in Figs. 3d, f, and 4, the AreaSum method is applied to this dataset. For the hierarchical-pair architecture used in Fig. 5, the AreaSum method is applied to the expression of 11,281 genes in 11,803 human cells. The gene list and hierarchical cluster are available in Supplementary Table 1.

### Learning hierarchical-pair model with an mRNA pool

In the model in Figs. 4 and 5, the hierarchical pairs are assumed to be signal transduction cascades to select a gene in an mRNA pool, similarly to the Monte Carlo tree search. At each repetition, a pair is stochastically chosen among pairs in the top seven layers depending on the coverage of the pair. In the selected pair, the competitive amplification is performed; branch *A* or *B* is selected at a ratio (*x*_*A*_ + *β*):(*x*_*B*_ + *β*), where bias *β* is 1 if not indicated to be 10^−7^, and the value of the selected branch, *x*_*A*_ or *x*_*B*_, increases by one. The downstream pair of the selected branch also enters the process of competitive amplification until the selected branch indicates a single gene. In an mRNA pool, mRNA of the selected gene increases by one, with randomly replacing one mRNA. Initially, 360,000 mRNAs in the mRNA pool are set based on the initial ratio, in addition to the ratios in each pair. The values in each pair decrease by *MSE*-dependent decay every 10 repetitions on average. The expression probability is calculated as an infinite product of ratios in all pairs that contain the gene. The code is available in Code File 4.

### Resource datasets

For bacterial genes (Fig. 2), RNAseq data from *Escherichia coli* (BWk3) (GSE96706) are used^15^. Among the 4296 genes, 4096 genes with expression values greater than 2^1.9^ under either culture condition in the dataset are selected. The genes are set to form a hierarchical-pair architecture using the order within the genome. GSM2538622 (1A), GSM2538631 (10A), and GSM2538649 (27A) are used as data without antibiotics, with kanamycin, and with ciprofloxacin, respectively.

For human early embryogenesis (Figs. 3 and 4), 20 differently-labeled cells are selected from three scRNA-seq datasets (E-MTAB-3929, GSM2257302, and GSE75748) for gene expression in preimplantation embryos, in vitro cultured embryonic stem cells, and the downstream early mesoderm and endoderm progenitors^16–18^. 'E3.1.443', 'E4.1.1', 'E5.1.26', 'E6.1.72', and 'E7.2.138' are selected from E-MTAB-3929. 'APS.p1c1r2', 'D2_25somitomere.p9c1r1', 'DLL1PXM.p8c1r1', 'Earlysomite.p10c2r8', 'H7hESC.p7c1r4', 'LatM.p3c1r1', 'MPS3.p5c1r1', 'Sclerotome.p2c1r1', and 'cDM.p4c1r1' are selected from GSM2257302. 'H9.00hb4s_001', 'H9.12h_001', 'H9.24h_013', 'H9.36h_001', 'H9.72h_001', and 'H9.96h_001' are selected from GSE75748. For the 16,921 genes expressed at more than 10 TPM in either cell, hierarchical clustering analyses are applied. For the test data, another scRNA-seq dataset of human preimplantation embryos is used (GSE36552) after merging the 16,921 genes with the gene names and assigning 0 as the expression level of non-annotated genes^19^. GSM896806, GSM896809, GSM922146, GSM922158, GSM922178, and GSM922194 are used for scRNA-seq data of the zygote, 2-cell, 4-cell, 8-cell, morula, and blastocyst stages, respectively. In Fig. 3c, 3 zygotes and 12 4-cell datasets are used.

To generate a common hierarchical-pair architecture in Fig. 5 and Supplementary Table 1, 13 scRNA-seq datasets of human tissues are used; 515 peripheral blood cells (GSE97531)^20^, 836 hematopoietic stem and progenitor cells in the bone marrow, spleen, and peripheral blood (GSE143567)^21^, 1567 trophoblast and stromal cells from the placenta (GSE89497)^28^, 559 cardiomyocytes (GSE95140_human)^24^, 2148 endometrium cells from the uterus (GSE111976)^26^, 766 renal cells from kidney biopsy (GSE160048_human)^22^, 91 fallopian tube epithelial cells (GSE132149_sc16)^27^, 2036 retina cells from the eyes (GSE133707_P1)^23^, 134 primordial germ cells from a female embryo at 10 weeks of gestation (GSM2295850) and from a male embryo at 25 weeks of gestation (GSM2306040)^29^, 372 in vitro-cultured primary myoblasts (GSE52529)^25^, 498 in vitro-cultured embryonic stem cells and early mesoderm progenitors (GSM2257302)^17^, 758 in vitro-cultured embryonic stem cells and endoderm progenitors (GSE75748_sc_time_course_ec)^18^, and 1529 cells from early preimplantation embryos (E-MTAB-3929)^16^. Gene names are used, if available, to integrate multiple datasets. If not available, the gene name is determined as ‘symbol’ using MyGene.py in the Python package. The code is written in a comment form in Code File 2. For the 11,281 genes successfully annotated in all 13 datasets (11,803 cells), gene expression ratios are recalculated by normalizing the total expression of the 11,281 genes to 1,000,000. The gene list and hierarchical clustering are available in Supplementary Table 1.

For the test data in Fig. 5, 39 single-cell datasets of the zygote, 2-cell, 4-cell, 8-cell, morula, and blastocyst stages in the preimplantation human embryo GSE36552^19^, 9 datasets of hematopoietic progenitors, including GMP, MLP, and lymphoid-primed multi-potential progenitors in the human cord blood of normal donors from GSE100618^30^, and 13 PBMCs from normal donors from GSE161901^31^ are collected. Gene expression ratios are recalculated by normalizing the total expression of 11,281 genes to 1,000,000. GSM896806, GSM896809, GSM922146, GSM922158, GSM922178, and GSM922194 are used for scRNA-seq data of the zygote, 2-cell, 4-cell, 8-cell, morula, and blastocyst stages, respectively^19^. For PBMCs, cell types are determined based on the high expression of *CD19* and *IGHM* (immunoglobulin heavy constant mu) for B lymphocytes, *TRBC2* (T cell receptor beta constant 2) for T lymphocytes, and *CD33* for myeloid cells. Among the test data, GSM2689351 (P5_E5_MLP) is used for MLP, GSM2689085 (P5_G11_GMP) for GMP, GSM4916527 (NormalDonor1_untreated_PBMC_027) for a B cell, GSM4916502 (NormalDonor1_untreated_PBMC_002) for a T cell, and GSM4916594 (NormalDonor1_untreated_PBMC_094) for a myeloid cell^30,31^. In the text, GSM2689298 (P3_F5_MLP) and GSM2689390 (P6_G6_MLP) are used for MLPs, GSM2689057 (P4_G9_GMP) and GSM2689102 (P6_C9_GMP) for GMP, GSM4916525 (NormalDonor1_untreated_PBMC_025) and GSM4916536 (NormalDonor1_untreated_PBMC_036) for B cells, and GSM4916573 (NormalDonor1_untreated_PBMC_073) and GSM4916783 (NormalDonor4_untreated_PBMC_001) for T cells, and GSM4916557 (NormalDonor1_untreated_PBMC_057) and GSM4916559 (NormalDonor1_untreated_PBMC_059) for myeloid cells^30,31^.

### Statistical analysis

Paired *t*-test was applied for statistical analysis to compare the results in 12 target ratios in Fig. 3c.

## Supplementary Information

Supplementary Information.

## Data Availability

All relevant data supporting the key findings of this study are available within the article and its Supplementary information files. Further information and requests for resources and reagents should be directed to and will be fulfilled by the lead contact, Tomoyuki YAMAGUCHI (t.yamaguchi@tokushukai.jp).

## Code availability

The codes generated in this study are provided as a supplementary file. They are available at GitHub (https://github.com/tyamaguc-tky/Learning_pair).
